# Six Years of COVID-19: Lessons from Epidemiology, Vaccination Campaigns, Clinical Risk Stratification, and Thromboembolic Surveillance

**DOI:** 10.3390/vaccines14070611

**Published:** 2026-07-14

**Authors:** Carmine Siniscalchi, Egidio Imbalzano, Manuela Basaglia, Nicoletta Cerundolo, Tiziana Meschi, Beatrice Prati, Alberto Parise, Antonio Nouvenne, Pierpaolo Di Micco

**Affiliations:** 1 Internal Medicine Department, Parma University Hospital, 43125 Parma, Italy; csiniscalchi84@gmail.com (C.S.); mbasaglia80@gmail.com (M.B.); ncerundolo@ao.pr.it (N.C.); tiziana.meschi@unipr.it (T.M.); bprati@ao.pr.it (B.P.); aparise@ao.pr.it (A.P.); antonio.nouvenne@unipr.it (A.N.); 2 Internal Medicine Department, Messina University Hospital, 98124 Messina, Italy; egidio.imbalzano@unime.it; 3 Internal Medicine Ward, Santa Maria delle Grazie Hospital, 80078 Pozzuoli, Naples, Italy

**Keywords:** COVID-19, SARS-CoV-2, vaccines, epidemiology, surveillance, frailty, venous thromboembolism, pulmonary embolism, thrombosis, long COVID, clinical risk stratification

## Abstract

Six years after the emergence of SARS-CoV-2, COVID-19 remains a dynamic public health challenge shaped by viral evolution, heterogeneous population immunity, changing vaccine strategies, and the long-term consequences of acute infection. Although the transition from pandemic emergency to endemic circulation has reduced the global burden of severe disease, COVID-19 continues to affect vulnerable populations, particularly older adults, frail patients, immunocompromised individuals, and subjects with multiple comorbidities. Vaccination has substantially modified the clinical course of infection, reducing hospitalization, intensive care admission, and mortality, while also reshaping surveillance priorities toward variant monitoring, vaccine effectiveness, waning immunity, breakthrough infections, and long COVID. At the same time, the pandemic has highlighted the need for integrated clinical risk stratification, including age, sex, frailty, inflammatory biomarkers, respiratory support requirements, and thromboembolic risk. Venous and arterial thrombotic complications have represented a key feature of severe COVID-19 and remain relevant for both acute management and post-discharge follow-up. This narrative review summarizes the main lessons learned from six years of COVID-19, focusing on epidemiology, vaccination campaigns, clinical risk assessment, thromboembolic complications, and surveillance strategies. Particular attention is given to the need for multidisciplinary and data-driven approaches capable of integrating virological, epidemiological, clinical, geriatric, and vascular medicine perspectives. Future COVID-19 surveillance should move beyond case counting and incorporate vaccine impact, population vulnerability, thrombotic and bleeding complications, long-term outcomes, and preparedness for emerging variants.

## 1. Introduction

The coronavirus disease 2019 (COVID-19) pandemic represents one of the most significant public health crises of the modern era. Since the identification of severe acute respiratory syndrome coronavirus 2 (SARS-CoV-2) in late 2019, the infection has affected hundreds of millions of individuals worldwide, causing profound consequences for healthcare systems, economies, and societies. During the early phases of the pandemic, uncertainty regarding viral transmission, clinical manifestations, prognostic factors, and effective therapeutic strategies contributed to unprecedented global morbidity and mortality. The rapid international spread of SARS-CoV-2 highlighted the vulnerability of healthcare infrastructures and emphasized the critical importance of coordinated epidemiological surveillance, timely public health interventions, and international scientific collaboration [1,2,3,4,5].

Over subsequent years, the epidemiological landscape of COVID-19 underwent substantial changes. The emergence of viral variants with distinct transmissibility, immune-evasion capabilities, and clinical characteristics continuously reshaped the course of the pandemic. Simultaneously, the large-scale implementation of vaccination campaigns dramatically reduced rates of severe disease, intensive care unit admission, and death, transforming COVID-19 from an acute global emergency into a more manageable, although still clinically relevant, infectious disease [6,7,8,9,10]. Nevertheless, persistent viral circulation, waning immunity, breakthrough infections, and the development of post-acute sequelae continue to pose important challenges for healthcare systems worldwide.

Beyond respiratory involvement, COVID-19 rapidly emerged as a complex multisystem disease characterized by endothelial dysfunction, immune dysregulation, inflammation, coagulation abnormalities, and thrombotic complications. Venous thromboembolism (VTE), including deep vein thrombosis and pulmonary embolism, became recognized as a major determinant of adverse outcomes, particularly among hospitalized and critically ill patients. Beyond thromboembolic complications, COVID-19 has been associated with a broad spectrum of extrapulmonary manifestations involving the cardiovascular, neurological, renal, gastrointestinal, endocrine, hematological, and immune systems. These manifestations may occur during the acute phase or persist after viral clearance, contributing to long-term morbidity, functional decline, and increased healthcare utilization. Therefore, COVID-19 should be considered a multisystem disease rather than a purely respiratory infection [11,12,13,14]. These observations stimulated extensive research into thromboprophylaxis strategies, risk stratification models, biomarker-guided approaches, and long-term vascular surveillance programs [11,12,13,14]. As knowledge accumulated, attention progressively shifted from acute infection management toward comprehensive assessment of long-term complications and individualized risk profiles.

The pandemic also underscored the importance of identifying vulnerable populations at increased risk of poor outcomes. Advanced age, multimorbidity, frailty, male sex, obesity, cardiovascular disease, chronic kidney disease, and immunosuppression consistently emerged as major prognostic determinants. More recently, studies have demonstrated the value of integrating traditional clinical variables with geriatric assessments, laboratory biomarkers, and functional measures to improve risk prediction and guide clinical decision-making. Such approaches have become increasingly relevant as the global population ages and the burden of chronic diseases continues to rise [15,16,17,18,19].

Six years after the emergence of SARS-CoV-2, important lessons can be drawn regarding epidemiological surveillance, vaccination strategies, clinical risk stratification, and thromboembolic monitoring. The experience gained during the pandemic has reshaped public health preparedness and provided valuable insights applicable not only to COVID-19 but also to future emerging infectious threats. The major epidemiological phases of the pandemic, dominant variants, public health responses, and their principal clinical implications are summarized in Table 1.

The aim of this narrative review is to provide a comprehensive overview of the principal lessons learned during six years of COVID-19, focusing on epidemiological trends, vaccination campaigns, clinical risk assessment, thromboembolic complications, and future surveillance strategies. Particular emphasis is placed on the integration of clinical, epidemiological, geriatric, and vascular medicine perspectives to support more effective management of current and future public health challenges.

### Literature Search Strategy

As this article was conceived as a narrative review, a formal systematic review methodology was not applied. Nevertheless, the literature search was conducted in a structured manner to identify the most relevant evidence published between January 2020 and June 2026. Electronic searches were performed in PubMed/MEDLINE using combinations of the following keywords: “COVID-19”, “SARS-CoV-2”, “vaccination”, “variants”, “long COVID”, “frailty”, “risk stratification”, “venous thromboembolism”, “pulmonary embolism”, “biomarkers”, “surveillance”, and “public health”. Additional relevant publications were identified through reference lists of retrieved articles and international guideline documents. Priority was given to randomized clinical trials, meta-analyses, large observational studies, and international guidelines considered relevant to the objectives of this review.

## 2. Epidemiological Evolution of COVID-19 from 2020 to 2026

The epidemiology of COVID-19 has undergone profound transformations since the first reported cases of SARS-CoV-2 infection in late 2019. The first cluster of atypical pneumonia was reported in Wuhan, Hubei Province, China, in December 2019, with early epidemiological links to the Huanan Seafood Wholesale Market [20]. Human-to-human transmission was subsequently confirmed, and the virus rapidly spread beyond China through international travel, with early imported cases reported in Thailand, Japan, South Korea, Europe, and North America. During February and March 2020, major outbreaks were recorded in Northern Italy, Spain, France, the United Kingdom, and the United States, marking the transition from regional epidemic clusters to sustained global transmission. On 11 March 2020, the World Health Organization declared COVID-19 a pandemic, reflecting the rapid spread of SARS-CoV-2 across continents and the growing burden on healthcare systems [21]. During the initial phase of the pandemic, the absence of population immunity, limited diagnostic capacity, and lack of effective therapeutic interventions contributed to rapid viral spread and substantial mortality worldwide. Early epidemiological studies demonstrated that transmission occurred efficiently through respiratory droplets and aerosols, allowing the virus to disseminate across continents within a few months. Consequently, healthcare systems faced an unprecedented burden characterized by hospital overcrowding, shortages of intensive care resources, and high case-fatality rates, particularly among older adults and patients with pre-existing comorbidities [22,23,24,25,26].

During 2020 and early 2021, non-pharmaceutical interventions, including lockdowns, social distancing measures, mask mandates, travel restrictions, and contact tracing programs, represented the primary tools available to limit viral transmission. Non-pharmaceutical interventions differed substantially across countries. China initially adopted strict containment measures, including lockdowns, mass testing, isolation of cases, quarantine of contacts, and travel restrictions. Italy was among the first European countries to implement a nationwide lockdown during the first wave, followed by several European countries that introduced school closures, stay-at-home orders, mask mandates, and restrictions on public gatherings. Other countries adopted different strategies, ranging from elimination-oriented approaches, as in New Zealand and Australia, to less restrictive mitigation strategies, as initially observed in Sweden. Comparative studies suggested that early and combined interventions were more effective than isolated measures, particularly when restrictions were implemented before widespread community transmission [27]. However, these measures also produced relevant social, psychological, educational, and economic consequences, including delayed medical care, mental health burden, reduced social interaction, and widening inequalities [28]. Thus, one of the major lessons from the pandemic is that non-pharmaceutical interventions may reduce viral transmission but should be timely, proportionate, clearly communicated, and accompanied by social and economic support [29]. Although these measures substantially reduced infection rates in many regions, their effectiveness varied according to timing, adherence, population density, socioeconomic factors, and healthcare infrastructure. Simultaneously, the rapid development of diagnostic testing platforms enabled more accurate case identification and facilitated the establishment of national and international surveillance networks [30,31,32,33].

The emergence of viral variants soon became a defining feature of the pandemic. The Alpha variant (B.1.1.7), first identified in the United Kingdom, demonstrated increased transmissibility compared with ancestral strains and rapidly became dominant in multiple countries. Subsequently, the Delta variant (B.1.617.2) was associated with even greater transmissibility, increased hospitalization rates, and higher healthcare utilization, resulting in major epidemic waves during 2021 despite expanding vaccination coverage. These observations highlighted the importance of continuous genomic surveillance and international data-sharing systems capable of rapidly identifying variants of concern [34,35,36,37].

A major epidemiological transition occurred with the emergence of the Omicron variant in late 2021. Omicron exhibited remarkable transmissibility and substantial immune-evasion capabilities, resulting in unprecedented numbers of infections globally. However, despite the dramatic increase in case incidence, population-level mortality rates progressively declined owing to widespread vaccine-induced immunity, previous natural infections, improved clinical management, and the intrinsically lower pathogenicity of some Omicron sublineages. Consequently, the relationship between infection incidence and severe clinical outcomes became progressively weaker than during earlier pandemic phases [37,38,39,40,41]. Main SARS-CoV-2 variants of concern and their principal epidemiological characteristics are presented in Table 2.

By 2023 and 2024, most countries had transitioned from emergency containment strategies toward long-term management approaches focused on protecting high-risk populations, maintaining vaccination programs, and monitoring healthcare system impact. Surveillance systems evolved accordingly, shifting emphasis from simple case counting toward the assessment of hospitalization rates, mortality, vaccine effectiveness, variant circulation, and long-term health consequences. Wastewater monitoring, genomic sequencing platforms, and integrated digital surveillance systems emerged as increasingly valuable tools for detecting epidemiological trends and anticipating future outbreaks [42,43,44].

**Figure 1:**
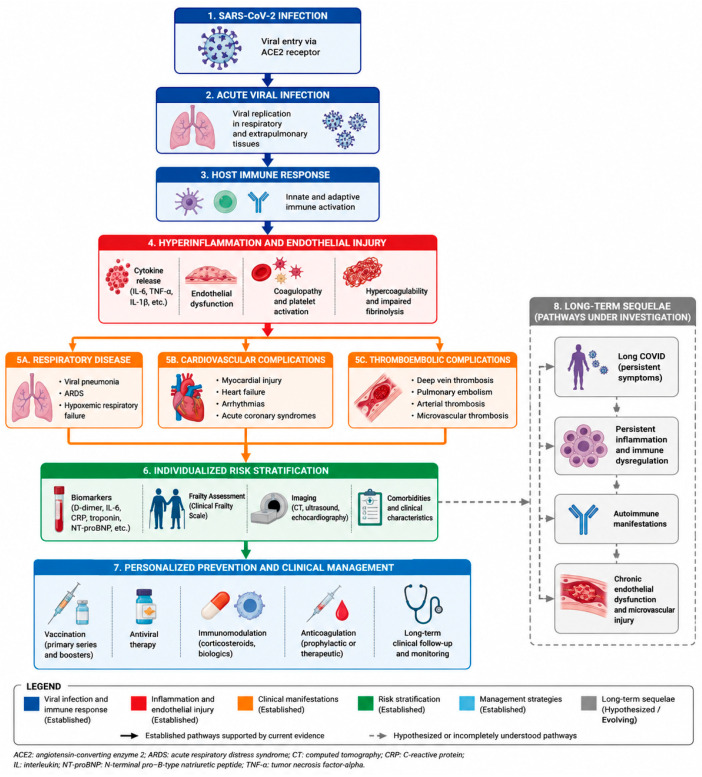
Integrated conceptual framework of COVID-19: from SARS-CoV-2 infection to personalized risk stratification, clinical management, and long-term sequelae. **Abbreviations:** ACE2, angiotensin-converting enzyme 2; ARDS, acute respiratory distress syndrome; CRP, C-reactive protein; CT, computed tomography; COVID-19, Coronavirus Disease 2019; IL, interleukin; NT-proBNP, N-terminal pro-B-type natriuretic peptide; SARS-CoV-2, Severe Acute Respiratory Syndrome Coronavirus 2; TNF-α, tumor necrosis factor alpha. **Legend.** Schematic overview of the principal pathophysiological mechanisms underlying COVID-19 and their clinical implications. SARS-CoV-2 infection triggers innate and adaptive immune responses that, in susceptible individuals, may progress to hyperinflammation, endothelial dysfunction, and coagulation abnormalities, resulting in respiratory, cardiovascular, and thromboembolic complications. These established mechanisms support individualized risk stratification based on biomarkers, frailty assessment, imaging findings, and comorbidities, guiding personalized preventive and therapeutic strategies, including vaccination, antiviral treatment, immunomodulation, anticoagulation, and long-term clinical follow-up. Continuous arrows indicate biological pathways consistently supported by current evidence, whereas dashed arrows indicate mechanisms that remain incompletely understood or are still under investigation, particularly those involved in long COVID, persistent inflammation, chronic endothelial dysfunction, and post-COVID autoimmune manifestations.

Although COVID-19 is currently characterized by endemic circulation in most regions of the world, the disease continues to exert a substantial burden on public health. Vulnerable populations, including frail older adults, immunocompromised patients, and individuals with chronic cardiovascular, respiratory, metabolic, or oncological conditions, remain at increased risk of severe outcomes. Furthermore, the persistence of long COVID syndromes, recurrent epidemic waves, and the possibility of novel variants underscore the continued importance of robust surveillance infrastructures. Figure 1 provides an integrated overview of COVID-19 pathophysiology, illustrating the progression from SARS-CoV-2 infection to immune activation, endothelial dysfunction, acute clinical manifestations, individualized risk stratification, personalized management, and long-term sequelae. During 2025 and 2026, widespread hybrid immunity resulting from previous infection and repeated vaccination has substantially reduced the incidence of severe disease despite continued viral circulation. Hospital admissions are now concentrated mainly among older adults, immunocompromised individuals, and patients with multiple chronic conditions, whereas repeated infections in immunocompetent individuals generally present with milder clinical manifestations. Nevertheless, recurrent infections continue to contribute to healthcare utilization and may increase the cumulative risk of long COVID in susceptible populations.

## 3. Vaccination Campaigns, Booster Strategies, and Vaccine Effectiveness

Natural SARS-CoV-2 infection induces both innate and adaptive immune responses [45]. During the early phase of infection, innate immunity contributes to viral control through interferon signaling, activation of macrophages and dendritic cells, natural killer cell responses, and production of inflammatory cytokines. In severe COVID-19, however, dysregulated innate immune activation may contribute to hyperinflammation, endothelial injury, and tissue damage. Adaptive immunity is characterized by the development of neutralizing antibodies directed mainly against the spike protein, together with CD4+ and CD8+ T-cell responses that contribute to viral clearance and long-term immune memory. The durability and quality of immune protection vary according to age, comorbidities, immunosuppression, viral variant, vaccination status, and previous infection history.

In addition to conventional neutralizing antibodies, recent experimental work has described antibodies capable of hydrolyzing the SARS-CoV-2 spike protein and spike-derived peptides, suggesting that catalytic antibody activity may represent an additional, although still investigational, mechanism involved in antiviral immune responses [46]. This observation broadens the understanding of humoral immunity beyond classical neutralization and supports further investigation into qualitative differences in antibody function after infection and vaccination.

The development of effective vaccines against SARS-CoV-2 represents one of the most remarkable achievements in the history of modern medicine. Less than one year after the identification of the viral genome, multiple vaccine platforms, including messenger RNA (mRNA), adenoviral vector, protein subunit, and inactivated virus vaccines, had completed large-scale clinical trials and received emergency authorization in several countries. This unprecedented scientific accomplishment resulted from extensive international collaboration, substantial public investment, innovative vaccine technologies, and accelerated regulatory pathways that maintained rigorous safety standards while addressing an urgent global health crisis [47,48,49,50,51,52].

The development of COVID-19 vaccines progressed through multiple technological platforms [53]. mRNA vaccines, including BNT162b2 and mRNA-1273, were designed to deliver genetic instructions encoding the prefusion-stabilized spike protein, inducing both humoral and cellular immune responses. Adenoviral vector vaccines, such as ChAdOx1 nCoV-19 and Ad26.COV2.S, used replication-defective viral vectors to deliver spike antigen information. Protein subunit vaccines provided purified antigenic components, often combined with adjuvants, whereas inactivated virus vaccines used chemically inactivated whole virions. The availability of different platforms allowed rapid global deployment but also revealed important differences in immunogenicity, storage requirements, durability of protection, reactogenicity, and adaptability to emerging variants.

Diagnostic testing evolved in parallel with vaccination and surveillance strategies. Reverse-transcription polymerase chain reaction (RT-PCR) represented the reference standard for detection of acute SARS-CoV-2 infection, particularly during the early pandemic phase [54]. Rapid antigen tests later enabled decentralized and repeated testing, although with lower sensitivity than molecular assays, especially in asymptomatic individuals or during low viral load phases. Serological assays were used to assess previous exposure, vaccine-induced antibody responses, and population-level seroprevalence, while neutralization assays and cellular immune tests provided more detailed information on functional immunity. Together, molecular, antigenic, and immunological diagnostics became essential tools for case detection, outbreak control, vaccine effectiveness studies, and long-term surveillance.

Initial vaccination campaigns prioritized healthcare workers, nursing home residents, older adults, and individuals at high risk of severe disease. Real-world evidence rapidly confirmed the efficacy observed in pivotal clinical trials, demonstrating substantial reductions in symptomatic infection, hospitalization, intensive care unit admission, and mortality. Countries achieving high vaccination coverage experienced marked declines in severe COVID-19 burden, even during periods of continued viral circulation. Importantly, vaccination not only protected vaccinated individuals but also contributed to reducing pressure on healthcare systems and preserving critical medical resources [52,53,54,55,56,57,58].

As the pandemic evolved, several challenges emerged that required adaptation of vaccination strategies. Waning immunity, breakthrough infections, and the appearance of novel variants with immune-evasive properties led to the implementation of booster vaccination programs. Numerous studies demonstrated that booster doses restored protection against severe disease and significantly reduced the risk of hospitalization and death, particularly among older adults and immunocompromised individuals. The emergence of Omicron and its sublineages highlighted the importance of maintaining flexible vaccination policies capable of responding to viral evolution and changing epidemiological conditions [59,60,61,62,63,64].

A critical lesson learned during successive vaccination campaigns was the importance of tailoring immunization strategies to vulnerable populations. Frail older adults, residents of long-term care facilities, patients with cancer, transplant recipients, and individuals receiving immunosuppressive therapies exhibited variable vaccine responses and often required additional booster doses or enhanced preventive measures. Consequently, risk-based vaccination approaches gradually replaced uniform population-wide strategies, allowing healthcare systems to allocate resources more efficiently while maximizing public health benefits [64,65,66,67,68].

The long-term effectiveness of COVID-19 vaccination should be evaluated not only in terms of infection prevention but also regarding mitigation of severe outcomes and long-term complications. Accumulating evidence suggests that vaccination reduces the incidence of long COVID, decreases persistent inflammatory responses, and may indirectly lower the risk of thromboembolic events associated with severe infection. Furthermore, the widespread adoption of vaccination substantially altered the clinical phenotype of COVID-19, contributing to the transition from a highly lethal pandemic disease toward a more manageable endemic respiratory infection [69,70,71,72].

By 2025 and 2026, vaccination policies increasingly focused on periodic booster administration for high-risk populations, similarly to established influenza vaccination programs. Several important questions remain unresolved, including the optimal frequency of booster administration, the duration of protection in different population groups, the potential impact of immune imprinting after repeated vaccination, and the role of next-generation vaccines capable of providing broader and more durable protection against future variants. Contemporary surveillance systems now integrate epidemiological, immunological, and genomic data to guide vaccine composition updates and identify populations requiring enhanced protection. These developments underscore the evolution of COVID-19 vaccination from an emergency intervention into a cornerstone of long-term public health preparedness. The principal COVID-19 vaccine platforms, their mechanisms of action, approximate effectiveness against severe disease, advantages, limitations, and current role in clinical practice are summarized in Table 3.

Although the overall effectiveness of vaccination is well established, uncertainty remains regarding the optimal booster schedule, the durability of protection in different populations, and the comparative effectiveness of updated vaccine formulations. Moreover, estimates of vaccine effectiveness have varied across studies according to circulating variants, previous immunity, age, and clinical outcomes considered.

## 4. Clinical Risk Stratification: Age, Frailty, Sex Differences, and Biomarkers

One of the most important lessons learned during the COVID-19 pandemic has been the recognition that the risk of severe disease is highly heterogeneous across individuals. Early epidemiological reports demonstrated substantial variability in clinical outcomes, ranging from asymptomatic infection to respiratory failure, multiorgan dysfunction, and death. This variability stimulated intense efforts to identify clinical characteristics capable of predicting adverse outcomes and supporting resource allocation during periods of healthcare system stress. Over time, age, frailty, sex, comorbidity burden, inflammatory biomarkers, and respiratory status emerged as some of the most robust determinants of prognosis [73,74,75,76,77].

Advanced age consistently represents the strongest risk factor for severe COVID-19 and mortality. Older adults exhibit immunosenescence, reduced physiological reserve, and a higher prevalence of chronic diseases that collectively increase vulnerability to acute infection. However, chronological age alone does not fully explain the marked heterogeneity observed among elderly patients. During the pandemic, increasing attention was directed toward frailty. Frailty is a multidimensional geriatric syndrome characterized by reduced physiological reserve [78], impaired homeostatic response, and increased vulnerability to stressors. In the context of acute infections such as COVID-19, frailty helps explain why individuals of the same chronological age may experience markedly different outcomes. Frail patients are more likely to develop respiratory deterioration, delirium, functional decline, prolonged hospitalization, institutionalization, and death after acute illness [79,80,81,82].

The incorporation of frailty assessments into routine clinical practice significantly improved risk stratification models. Instruments such as the Clinical Frailty Scale (CFS) proved particularly valuable because of their simplicity and prognostic accuracy. The Clinical Frailty Scale is a 9-point global clinical judgment tool that classifies patients from 1, corresponding to very fit individuals, to 9, corresponding to terminally ill patients. Intermediate categories describe increasing degrees of vulnerability, dependence in instrumental or basic activities of daily living, reduced mobility, and need for assistance. During the COVID-19 pandemic, the CFS was widely used because it is rapid, bedside-applicable, and able to summarize functional reserve and biological vulnerability beyond chronological age alone. Nevertheless, CFS assessment should reflect the patient’s baseline pre-illness status rather than the acute severity of COVID-19 at hospital presentation. More recently, comparative analyses conducted across different pandemic waves have shown that frailty retained a strong prognostic value even after widespread vaccination and improvements in clinical management. These findings suggest that frailty reflects biological vulnerability that extends beyond the specific characteristics of individual viral variants and remains relevant throughout the evolution of the pandemic [83].

Sex-related differences also emerged as important determinants of COVID-19 outcomes. From the earliest stages of the pandemic, men consistently exhibited higher rates of hospitalization, intensive care admission, thrombotic complications, and mortality compared with women. Several biological mechanisms have been proposed to explain these observations, including differences in immune responses, sex hormone regulation, endothelial function, inflammatory pathways, and expression of viral entry receptors. In addition, behavioral and socioeconomic factors may contribute to sex-specific patterns of disease severity and healthcare utilization [84,85,86,87,88].

Accumulating evidence has further demonstrated that sex differences persist even after adjustment for age and comorbidity burden. Recent analyses from large real-world cohorts have confirmed that sex remains an independent determinant of clinical outcomes across multiple pandemic phases. Understanding these differences may facilitate the development of more personalized preventive and therapeutic strategies while improving identification of individuals at increased risk of adverse outcomes [88].

Alongside demographic and functional characteristics, laboratory biomarkers became essential tools for risk stratification. Elevated concentrations of C-reactive protein, ferritin, interleukin-6, lactate dehydrogenase, cardiac troponins, and D-dimer were repeatedly associated with disease severity and mortality. Among these biomarkers, D-dimer attracted particular interest because of its close relationship with thromboinflammatory processes and venous thromboembolism. Elevated D-dimer levels were consistently associated with increased mortality risk, thrombotic complications, and need for advanced respiratory support [89,90,91,92].

Beyond classical inflammatory and coagulation biomarkers, several additional laboratory parameters have been investigated as potential predictors of COVID-19 severity [93]. Lymphopenia, neutrophil-to-lymphocyte ratio, platelet-to-lymphocyte ratio, procalcitonin, soluble urokinase plasminogen activator receptor, complement activation markers, markers of neutrophil extracellular traps, cardiac troponins, natriuretic peptides, and renal or hepatic injury markers have all been associated with disease severity or adverse outcomes in different cohorts. These biomarkers reflect distinct but interconnected mechanisms, including viral replication, immune dysregulation, endothelial injury, hypercoagulability, organ damage, and secondary bacterial infection.

MicroRNAs have also emerged as promising investigational biomarkers in COVID-19. These small non-coding RNAs regulate gene expression and may influence antiviral immunity, inflammation, endothelial activation, coagulation pathways, and tissue repair. Several studies have reported altered circulating microRNA profiles in patients with severe COVID-19, suggesting potential roles in prognosis, disease monitoring, and identification of molecular pathways involved in hyperinflammation and vascular injury. However, microRNA-based biomarkers remain investigational and require analytical standardization, external validation, and prospective evaluation before clinical implementation [94].

The interaction between respiratory failure and thrombotic risk also became increasingly evident as clinical experience accumulated. Patients requiring non-invasive ventilation or intensive respiratory support frequently exhibited higher rates of venous thromboembolism, reflecting the complex interplay between inflammation, endothelial injury, immobility, and hypercoagulability. These observations reinforced the need for integrated clinical assessment models capable of simultaneously evaluating respiratory status, frailty, biomarkers, and thrombotic risk [95].

Collectively, these findings support a multidimensional approach to COVID-19 risk stratification. Rather than relying on isolated clinical variables, contemporary surveillance and management strategies increasingly integrate demographic characteristics, functional status, biological markers, and disease severity indicators. Figure 1 provides an integrated overview of COVID-19 pathophysiology, illustrating the progression from SARS-CoV-2 infection to immune activation, endothelial dysfunction, acute clinical manifestations, individualized risk stratification, personalized management, and long-term sequelae.

Although numerous biomarkers have shown prognostic value, their optimal integration into routine clinical decision-making remains uncertain because of differences in study populations, assay standardization, timing of measurement, and outcome definitions.

## 5. Thromboembolic Complications and Antithrombotic Surveillance

Among the numerous clinical manifestations of COVID-19, thromboembolic complications rapidly emerged as one of the most distinctive and clinically relevant features of severe disease. During the first months of the pandemic, physicians observed unexpectedly high rates of venous and arterial thrombosis despite standard thromboprophylaxis protocols. Subsequent studies demonstrated that SARS-CoV-2 infection induces a unique thromboinflammatory state characterized by endothelial injury, platelet activation, cytokine-mediated coagulation activation, complement dysregulation, and microvascular thrombosis. This pathophysiological framework, often referred to as COVID-19-associated coagulopathy, fundamentally changed the clinical management of hospitalized patients and stimulated an unprecedented volume of research in vascular medicine and thrombosis [96,97,98,99,100].

Venous thromboembolism (VTE), including deep vein thrombosis (DVT) and pulmonary embolism (PE), became one of the most frequently reported complications among hospitalized patients. The incidence was particularly high among critically ill individuals requiring intensive care support, where thrombotic rates frequently exceeded those observed in other severe respiratory infections. Importantly, pulmonary thrombosis in COVID-19 appeared to result not only from classic embolic mechanisms but also from local immunothrombotic processes occurring within the pulmonary microvasculature. These observations expanded traditional concepts of venous thromboembolism and highlighted the close relationship between inflammation and thrombosis [101,102,103,104].

The identification of reliable predictors of thrombotic complications became a major priority. Elevated D-dimer concentrations consistently emerged as one of the strongest prognostic biomarkers, showing associations with disease severity, thrombotic events, respiratory failure, and mortality. Consequently, D-dimer measurements were incorporated into numerous clinical algorithms and surveillance strategies. More recently, age-adjusted D-dimer thresholds have been proposed to improve diagnostic accuracy and reduce unnecessary imaging procedures, particularly among older patients in whom baseline D-dimer values are frequently elevated [105,106,107].

As evidence accumulated, several randomized clinical trials evaluated different anticoagulation regimens in patients with COVID-19. While standard thromboprophylaxis remained appropriate for many hospitalized individuals, selected patient groups appeared to benefit from intensified anticoagulation strategies. Nevertheless, the balance between thrombotic prevention and bleeding risk remained challenging, particularly among older adults and patients with multiple comorbidities. These findings emphasized the importance of individualized risk assessment rather than universal escalation of anticoagulant therapy [108,109,110,111].

The pandemic also stimulated interest in adjunctive therapies that may interact with thromboinflammatory pathways. Statins, widely used for cardiovascular prevention, have been investigated because of their pleiotropic effects on endothelial function, inflammatory signaling, and coagulation processes. While their primary indication remains lipid lowering, several experimental and observational studies have explored their potential influence on vascular and thrombotic mechanisms. However, the extent to which these effects translate into clinically meaningful benefits in patients with COVID-19 or other thromboinflammatory conditions remains an area of ongoing research [112,113,114].

Evidence generated from large international registries has further strengthened interest in the potential vascular benefits of statins. Studies demonstrated significant associations between statin use and improved clinical outcomes among patients with venous thromboembolism, including those receiving anticoagulant therapy, individuals with acute pulmonary embolism, and patients with deep vein thrombosis [115,116]. Collectively, these studies suggest that modulation of thromboinflammatory pathways may have important implications for both COVID-19-related thrombosis and broader vascular disease management [117,118].

As the pandemic evolved, surveillance priorities progressively shifted from acute thrombotic event detection toward comprehensive vascular monitoring. Contemporary surveillance strategies increasingly incorporate clinical assessment, biomarker evaluation, imaging when indicated, bleeding risk estimation, and long-term follow-up of patients with thrombotic complications. This multidimensional approach has become particularly relevant in older and medically complex populations, where thrombotic and hemorrhagic risks frequently coexist. Figure 1 provides an integrated overview of COVID-19 pathophysiology, illustrating the progression from SARS-CoV-2 infection to immune activation, endothelial dysfunction, acute clinical manifestations, individualized risk stratification, personalized management, and long-term sequelae.

## 6. Long-Term Surveillance, Long COVID, and Future Preparedness

As the acute phase of the COVID-19 pandemic gradually transitioned toward endemic circulation, attention increasingly shifted from immediate infection control toward long-term surveillance and the management of persistent health consequences. Although vaccination campaigns and improved clinical management substantially reduced rates of hospitalization and mortality, SARS-CoV-2 continues to exert a considerable burden on healthcare systems worldwide. Consequently, surveillance strategies have evolved from simple case counting toward more comprehensive approaches that integrate epidemiological, clinical, immunological, and healthcare utilization data [119].

One of the most important challenges emerging during the later phases of the pandemic has been the recognition of post-acute sequelae of SARS-CoV-2 infection, commonly referred to as long COVID or post-COVID condition. According to commonly used definitions, symptoms may persist or newly appear several weeks after acute infection and continue for at least 2–3 months without an alternative explanation. Reported manifestations include fatigue, exertional intolerance, dyspnea, chest pain, palpitations, cognitive dysfunction or “brain fog”, sleep disturbances, headache, anosmia or dysgeusia, autonomic symptoms, musculoskeletal pain, anxiety, depression, and reduced quality of life. Symptoms may fluctuate over time and can persist for 6, 12, or even more than 24 months in a subset of patients [120].

Medical care for long COVID has generally required a multidisciplinary approach, including primary care physicians, internists, pulmonologists, cardiologists, neurologists, rehabilitation specialists, psychologists, and physiotherapists [121]. Management is mainly supportive and symptom-oriented, with attention to exclusion of alternative diagnoses, assessment of cardiopulmonary function, rehabilitation when appropriate, treatment of dysautonomia or sleep disorders, psychological support, and graded return to daily activities. However, excessive exertion may worsen symptoms in patients with post-exertional malaise, and individualized rehabilitation plans are therefore required. Dedicated long COVID clinics have been established in many countries, although access remains heterogeneous and evidence-based therapeutic options are still limited [122].

The mechanisms underlying long COVID remain incompletely understood and are likely multifactorial. Proposed pathophysiological pathways include persistent immune activation, chronic inflammation, viral persistence, endothelial dysfunction, autonomic dysregulation, microvascular injury, and residual organ damage. Importantly, many of these mechanisms overlap with those implicated in acute COVID-19-associated coagulopathy, further supporting the concept that vascular dysfunction may contribute not only to acute thrombotic complications but also to long-term clinical sequelae [123].

Surveillance programs implemented during recent years have highlighted the importance of identifying vulnerable populations requiring prolonged follow-up. Frail older adults, patients with cardiovascular disease, individuals with chronic respiratory disorders, and immunocompromised subjects remain disproportionately affected by both acute and long-term consequences of SARS-CoV-2 infection. The pandemic has therefore reinforced the value of multidimensional assessment models capable of integrating frailty, biological age, functional status, comorbidity burden, and social determinants of health into surveillance frameworks. Such approaches are particularly relevant in aging populations, where resilience to infectious threats may be substantially reduced [124].

Beyond patient-level monitoring, future preparedness efforts must focus on strengthening surveillance infrastructures capable of rapidly detecting emerging variants and novel infectious threats. The COVID-19 experience demonstrated the importance of genomic surveillance, wastewater monitoring, digital health platforms, integrated electronic health records, and international data-sharing networks. These systems enable timely identification of epidemiological changes and facilitate evidence-based public health responses. Moreover, advances in artificial intelligence and predictive analytics may further enhance the ability to identify high-risk populations, anticipate healthcare needs, and optimize resource allocation during future outbreaks [125].

Another critical lesson concerns the importance of maintaining public confidence in vaccination programs and scientific institutions. Vaccine hesitancy, misinformation, and disparities in healthcare access significantly influenced pandemic outcomes across different regions. Future preparedness strategies must therefore combine scientific innovation with effective communication, equitable healthcare delivery, and transparent public health policies. Investments in vaccine research, surveillance infrastructure, and international collaboration remain essential to mitigate the impact of future pandemics [126].

Six years after the emergence of SARS-CoV-2, it has become evident that sustainable surveillance systems must extend beyond the monitoring of infection incidence alone. Contemporary approaches should incorporate vaccine effectiveness, long COVID burden, thrombotic and bleeding complications, healthcare utilization, and population vulnerability. Such multidimensional surveillance frameworks are likely to represent one of the most enduring public health legacies of the COVID-19 pandemic and may serve as a model for future infectious disease preparedness initiatives [127].

Another important long-term consequence of SARS-CoV-2 infection is the possible development or unmasking of autoimmune phenomena. Several mechanisms have been proposed, including molecular mimicry, bystander immune activation, epitope spreading, persistent inflammation, dysregulated interferon responses, and disruption of immune tolerance [128]. Autoantibodies targeting phospholipids, interferons, nuclear antigens, endothelial structures, and other immune-related targets have been described in subsets of patients with COVID-19. Clinically, SARS-CoV-2 infection has been associated with immune-mediated manifestations such as Guillain–Barré syndrome, autoimmune cytopenias, thyroiditis, vasculitis, inflammatory arthritis, systemic lupus erythematosus-like syndromes, and new-onset or relapsing autoimmune diseases. Although causality remains difficult to establish in many cases, these observations suggest that post-COVID surveillance should include attention to persistent inflammatory, vascular, and autoimmune manifestations, particularly in patients with compatible symptoms.

Likewise, considerable heterogeneity persists regarding the definition, pathophysiology, and optimal management of long COVID, highlighting the need for standardized diagnostic criteria and prospective studies.

## 7. Future Perspectives: From Pandemic Response to Integrated Infectious Disease Surveillance

The COVID-19 pandemic has fundamentally transformed the way healthcare systems approach infectious disease surveillance, public health preparedness, and risk assessment. While the initial response focused primarily on limiting viral transmission and reducing mortality, the experience accumulated over six years has demonstrated the need for more comprehensive surveillance models capable of integrating epidemiological, clinical, laboratory, and societal data. Future preparedness efforts should therefore move beyond traditional infectious disease monitoring and embrace multidimensional frameworks that capture the full spectrum of disease burden and population vulnerability.

The key lessons learned from six years of COVID-19 can be summarized across four major domains. First, healthcare systems represented a major bottleneck during the emergency phase [129]. Beyond surveillance, future preparedness also requires sustainable healthcare resource planning, economic evaluation of preventive strategies, and incorporation of Health Technology Assessment (HTA) into pandemic preparedness. Cost-effectiveness analyses may help optimize vaccination strategies, antiviral use, diagnostic testing, and allocation of healthcare resources, particularly during periods of limited availability. Shortages of hospital beds, intensive care capacity, trained personnel, personal protective equipment, and diagnostic resources directly influenced outcomes and highlighted the need for sustained investment in hospital preparedness and surge capacity. Second, digital and online tools proved essential [130]. Telemedicine, electronic health records, digital surveillance platforms, remote monitoring, online education, and data-sharing infrastructures helped maintain healthcare continuity and accelerated epidemiological response. Third, the pandemic demonstrated the extraordinary potential of scientific progress [131]. SARS-CoV-2 became one of the most intensively studied viruses in history, leading to the rapid development of diagnostics, vaccines, antiviral strategies, immunomodulatory treatments, genomic surveillance platforms, and large collaborative clinical trials. Fourth, the pandemic exposed and worsened social inequalities [132]. Disadvantaged communities experienced disproportionate infection risk, reduced access to healthcare, higher occupational exposure, lower vaccine access or uptake, and greater socioeconomic consequences from containment measures. Future preparedness must therefore integrate social protection, equitable access to basic healthcare services, transparent communication, and targeted protection of vulnerable populations. Table 4 summarizes the principal lessons learned from the COVID-19 pandemic and their implications for future infectious disease preparedness.

One of the most important advances emerging from the pandemic has been the widespread implementation of real-time surveillance infrastructures. Genomic sequencing networks, wastewater monitoring programs, digital reporting systems, and integrated electronic health records have significantly enhanced the ability to detect epidemiological changes and identify emerging variants. These systems have demonstrated that timely access to high-quality data is essential for guiding evidence-based decision-making and implementing effective public health interventions. Future surveillance programs should maintain and expand these capabilities, ensuring rapid responses to emerging pathogens while preserving interoperability across national and international healthcare systems.

Another critical lesson concerns the importance of integrating clinical vulnerability into surveillance frameworks. Traditional epidemiological indicators such as incidence rates and mortality statistics provide valuable information but often fail to identify populations at highest risk of adverse outcomes. The COVID-19 experience demonstrated that age, frailty, comorbidity burden, sex-specific factors, immunological status, and socioeconomic determinants substantially influence disease trajectories. Consequently, future surveillance systems should incorporate measures of biological vulnerability alongside conventional epidemiological metrics to improve risk prediction and resource allocation.

The increasing availability of large-scale healthcare databases and artificial intelligence technologies offers additional opportunities for improving surveillance strategies. Machine-learning algorithms may facilitate early identification of high-risk individuals, prediction of healthcare utilization, detection of emerging epidemiological trends, and optimization of vaccination programs. Similarly, digital health platforms and remote monitoring systems may enable more personalized approaches to patient follow-up, particularly among older adults and individuals with chronic diseases. Such innovations have the potential to enhance both healthcare efficiency and patient outcomes while reducing the burden on healthcare infrastructures.

The pandemic also highlighted the importance of maintaining strong international scientific collaborations. Large multinational registries, collaborative clinical trials, and global surveillance initiatives generated high-quality evidence at an unprecedented pace. These collaborative networks played a pivotal role in advancing understanding of COVID-19 pathophysiology, vaccine effectiveness, thrombotic complications, and long-term outcomes. Preserving and strengthening these international partnerships will be essential for addressing future infectious disease threats and ensuring rapid dissemination of scientific knowledge.

Finally, the long-term consequences of COVID-19 underscore the need for surveillance systems capable of monitoring not only acute infections but also chronic and delayed health outcomes. Long COVID, thromboembolic complications, functional decline, and persistent cardiovascular sequelae have demonstrated that the burden of infectious diseases frequently extends far beyond the acute phase. Future surveillance models should therefore integrate short-term and long-term outcome assessment, facilitating a more comprehensive understanding of disease impact and supporting the development of targeted preventive strategies.

The transition from pandemic emergency management to sustainable infectious disease surveillance represents one of the most important public health challenges of the coming decade. The lessons learned from COVID-19 provide a unique opportunity to develop more resilient, data-driven, and patient-centered surveillance systems capable of responding effectively to future global health threats.

## 8. Strengths and Limitations of Current Evidence

This review has several strengths. First, it provides an integrated overview of six years of COVID-19 research by combining epidemiological, vaccinological, clinical, geriatric, and thromboembolic perspectives within a single framework. While many reviews have focused on individual aspects of the pandemic, the present work aims to summarize the most relevant lessons learned across multiple disciplines, emphasizing the interactions among viral evolution, vaccination strategies, population vulnerability, clinical risk stratification, and vascular complications. Second, particular attention has been devoted to vulnerable populations, including older adults, frail individuals, and patients with chronic diseases, groups that continue to experience a disproportionate burden of severe outcomes despite the transition toward endemic circulation. Third, the review highlights the importance of thromboembolic surveillance and individualized risk assessment, topics that remain highly relevant for both acute COVID-19 management and long-term follow-up.

Several limitations should also be acknowledged. First, this is a narrative review and therefore does not follow the methodological rigor of a systematic review or meta-analysis. Consequently, the selection of studies may be influenced by publication bias and by the availability of evidence at the time of manuscript preparation. Second, the scientific literature on COVID-19 continues to evolve rapidly, particularly regarding emerging variants, updated vaccine formulations, long COVID, and novel surveillance strategies. As a result, some findings may require future revision as new evidence becomes available. Third, considerable heterogeneity exists among published studies with respect to patient populations, healthcare settings, definitions of outcomes, vaccination status, and circulating viral variants, potentially limiting direct comparisons across investigations. Finally, most available evidence originates from high-income countries with well-developed surveillance infrastructures, which may reduce the generalizability of some observations to low- and middle-income settings.

Despite these limitations, the large body of evidence accumulated during the pandemic provides a robust foundation for understanding the epidemiological evolution of COVID-19 and for improving preparedness for future infectious disease threats. Continued surveillance, international collaboration, and periodic reassessment of emerging evidence will remain essential to refine prevention strategies, optimize risk stratification models, and protect vulnerable populations.

## 9. Conclusions

Six years after the emergence of SARS-CoV-2, COVID-19 continues to provide valuable lessons for clinicians, researchers, and public health authorities worldwide. The pandemic has demonstrated the critical importance of timely epidemiological surveillance, rapid vaccine development, international scientific collaboration, and evidence-based public health interventions. At the same time, it has revealed the complex interplay between infection, inflammation, thrombosis, aging, frailty, and chronic disease, emphasizing the need for multidimensional approaches to risk assessment and patient management.

Vaccination has profoundly altered the natural history of COVID-19, reducing severe disease and mortality while facilitating the transition from an acute global emergency toward endemic circulation. Nevertheless, persistent viral evolution, long COVID, and the continued vulnerability of frail and medically complex populations highlight the need for ongoing surveillance and preventive strategies. The pandemic has also underscored the central role of thromboembolic complications in determining clinical outcomes and has stimulated important advances in antithrombotic management and vascular surveillance.

Looking ahead, future preparedness efforts should build upon the infrastructures, technologies, and collaborative networks developed during the COVID-19 era. Integrated surveillance systems combining epidemiological, clinical, genomic, laboratory, and digital health data may provide a more accurate representation of disease burden and facilitate more effective public health responses. Particular attention should be directed toward vulnerable populations, including older adults, frail individuals, and patients with chronic diseases, who remain disproportionately affected by infectious threats.

Several important research priorities remain. These include improving the understanding of long-term immunity, the clinical consequences of repeated SARS-CoV-2 infections, optimization of booster vaccination strategies, development of precision vaccination approaches for vulnerable populations, identification of effective therapies for long COVID, and integration of artificial intelligence and real-world evidence into future surveillance systems.

Ultimately, the legacy of COVID-19 extends beyond the management of a single disease. The experience gained during the pandemic offers a framework for addressing future global health emergencies through coordinated surveillance, personalized risk stratification, equitable vaccination strategies, and sustained international collaboration. These lessons will remain relevant long after the pandemic itself, shaping the future of infectious disease prevention, monitoring, and clinical care.

## Figures and Tables

**Table 1:** Evolution of the COVID-19 pandemic: major epidemiological phases, dominant variants, public health responses, and clinical implications.

Pandemic Phase	Approximate Period	Dominant Variant(s)	Key Epidemiological Characteristics	Main Public Health Response	Principal Clinical Implications
Emergence and global spread	Dec 2019–Feb 2020	Ancestral Wuhan strain	Initial outbreaks in China followed by rapid international dissemination; WHO declared a pandemic on 11 March 2020	Case identification, isolation, travel restrictions, early surveillance	Severe viral pneumonia, high case fatality, absence of vaccines or specific therapies
First pandemic waves	Mar 2020–Dec 2020	Ancestral strain	Exponential growth of infections and hospitalizations; repeated epidemic waves worldwide	National lockdowns, physical distancing, universal masking, contact tracing, expansion of diagnostic testing	High burden on healthcare systems; recognition of thromboembolic complications and multisystem disease
Vaccination era	2021	Alpha, Beta, Gamma, Delta	Progressive increase in vaccine coverage; Delta associated with increased transmissibility and hospitalization	Mass vaccination campaigns, booster dose introduction, genomic surveillance	Marked reduction in severe disease, intensive care admissions, and mortality despite continued viral circulation
Omicron transition	Late 2021–2023	Omicron and sublineages	Extremely high transmissibility with lower average severity in immunized populations; increasing hybrid immunity	Updated booster campaigns, targeted vaccination of vulnerable populations, adaptation of surveillance systems	Declining disease severity; increasing importance of long COVID and management of high-risk patients
Endemic circulation	2024–2026	Omicron descendants (e.g., JN.1 and related sublineages)	Sustained viral circulation with seasonal fluctuations; repeated infections common; widespread hybrid immunity	Risk-based vaccination strategies, integrated genomic surveillance, long-term monitoring	Hospitalizations concentrated in frail, elderly, and immunocompromised individuals; emphasis on personalized prevention, long COVID management, and healthcare preparedness

Abbreviations: WHO, World Health Organization. Legend: Major epidemiological phases of the COVID-19 pandemic from its emergence to the current endemic phase. The table summarizes the dominant viral variants, principal epidemiological characteristics, public health responses, and major clinical implications that have shaped the evolution of SARS-CoV-2 over the past six years. Epidemiological characteristics and clinical impact varied according to population immunity, vaccination coverage, healthcare capacity, and circulating viral variants.

**Table 2:** Main SARS-CoV-2 variants of concern, key spike mutations, epidemiological impact, and public health implications.

Variant	First Detection/Period of Dominance	Key Spike Mutations	Main Epidemiological Features	Public Health Implications
Alpha (B.1.1.7)	First detected in the United Kingdom in late 2020; dominant in early 2021	N501Y, Δ69–70, P681H	Increased transmissibility compared with ancestral strains	Reinforced the need for genomic surveillance and rapid public health responses
Beta (B.1.351)	First detected in South Africa in 2020	K417N, E484K, N501Y	Immune escape and reduced neutralization by some antibodies	Highlighted the importance of monitoring vaccine effectiveness against variants
Gamma (P.1)	First detected in Brazil/Japan travelers in late 2020–early 2021	K417T, E484K, N501Y	Increased transmissibility and immune escape	Raised concerns regarding reinfections in previously exposed populations
Delta (B.1.617.2)	First detected in India in late 2020; globally dominant in 2021	L452R, T478K, P681R	Markedly increased transmissibility and higher hospitalization risk than previous variants	Caused major epidemic waves despite increasing vaccine coverage
Omicron (B.1.1.529 and sublineages)	First reported in Southern Africa in late 2021; dominant from 2022 onward	Multiple spike mutations, including N501Y, Q498R, E484A, K417N, P681H, H655Y	Very high transmissibility and substantial immune escape, with lower average severity than Delta in highly immune populations	Shifted surveillance priorities toward booster strategies, immune escape, and protection of vulnerable groups

**Legend.** Main SARS-CoV-2 variants of concern and their principal epidemiological characteristics. The listed mutations are representative spike substitutions or deletions associated with altered transmissibility, receptor binding, immune escape, or furin cleavage efficiency. The clinical impact of each variant depended not only on intrinsic viral properties but also on population immunity, vaccination coverage, prior infection, healthcare capacity, and public health measures.

**Table 3:** Main COVID-19 vaccine platforms: mechanisms, effectiveness, advantages, limitations, and current role.

Vaccine Platform	Representative Vaccines	Mechanism of Action	Approximate Effectiveness Against Severe COVID-19 *	Main Advantages	Main Limitations	Current Role (2025–2026)
mRNA vaccines	BNT162b2 (Pfizer-BioNTech), mRNA-1273 (Moderna)	Lipid nanoparticle-delivered mRNA encoding the prefusion spike protein, inducing humoral and cellular immunity	>90% against hospitalization and death during the ancestral and early variant phases; maintained high protection after booster doses despite reduced protection against infection with Omicron	High immunogenicity, rapid manufacturing, easy adaptation to emerging variants	Waning protection against infection; booster doses required; cold-chain storage	Preferred platform for booster vaccination and protection of high-risk populations
Viral vector vaccines	ChAdOx1 nCoV-19 (Oxford–AstraZeneca), Ad26.COV2.S (Johnson & Johnson)	Replication-defective adenoviral vectors expressing the spike protein	Approximately 70–90% against severe disease during early pandemic phases	Easier storage and distribution; strong cellular immune response	Rare vaccine-induced immune thrombotic thrombocytopenia (VITT); reduced use in many countries	Limited use; largely replaced by updated mRNA vaccines in high-income settings
Protein subunit vaccines	NVX-CoV2373 (Novavax)	Recombinant spike protein with adjuvant	Approximately 80–90% against severe disease	Favorable safety profile; alternative for individuals unable to receive mRNA vaccines	Slower production; limited real-world experience compared with mRNA vaccines	Alternative option in selected populations
Inactivated whole-virus vaccines	CoronaVac (Sinovac), BBIBP-CorV (Sinopharm)	Chemically inactivated whole SARS-CoV-2 virus	Approximately 60–80%; lower effectiveness in older adults without boosters	Established manufacturing technology; easier implementation in many countries	Lower immunogenicity; more rapid waning of protection	Continued use in several low- and middle-income countries, generally with booster strategies
Updated variant-adapted vaccines	XBB-based and subsequent updated formulations	Modified antigen targeting recently circulating variants	Improved neutralizing activity against contemporary variants; protection against severe disease remains high	Better match with circulating variants; improved booster responses	Frequent antigen updates may be required because of viral evolution	Central component of current booster strategies for vulnerable populations

* Approximate effectiveness estimates refer primarily to protection against severe COVID-19, hospitalization, and death, rather than protection against SARS-CoV-2 infection, and should be interpreted considering differences in circulating variants, booster status, and population characteristics. **Abbreviations:** mRNA, messenger RNA; VITT, vaccine-induced immune thrombotic thrombocytopenia; COVID-19, Coronavirus Disease 2019. **Legend.** Comparison of the principal COVID-19 vaccine platforms according to mechanism of action, representative products, approximate effectiveness against severe disease, major advantages and limitations, and current role in clinical practice. Reported effectiveness estimates are approximate and derived from pivotal randomized clinical trials and large real-world effectiveness studies. Actual effectiveness varies according to circulating variants, previous infection, vaccination schedule, booster administration, age, and host characteristics.

**Table 4:** Key lessons learned from six years of COVID-19 and implications for future preparedness.

Domain	Lesson Learned	Implication for Future Preparedness
Healthcare systems	Hospitals, ICUs, personnel, and supply chains represented critical bottlenecks	Invest in surge capacity, workforce planning, flexible hospital organization, and essential medical supplies
Digital infrastructure	Telemedicine, electronic records, digital surveillance, and online communication became essential tools	Maintain interoperable digital platforms for surveillance, clinical care, and rapid information exchange
Scientific progress	SARS-CoV-2 became one of the most studied viruses, accelerating diagnostics, vaccines, therapeutics, and genomic surveillance	Preserve research networks, adaptive trial platforms, and rapid regulatory pathways
Vaccination	Rapid vaccine development reduced severe disease, hospitalization, and mortality	Maintain flexible vaccine platforms and targeted booster strategies for vulnerable populations
Social inequalities	The pandemic worsened disparities in exposure, healthcare access, education, income, and vaccine uptake	Integrate social protection, equitable healthcare access, and targeted community interventions
Vulnerable populations	Older adults, frail patients, immunocompromised individuals, and multimorbid patients remained at highest risk	Incorporate frailty, comorbidity, and social determinants into risk stratification and surveillance
Long-term outcomes	Long COVID, functional decline, autoimmune phenomena, and thromboembolic sequelae extended the disease burden beyond acute infection	Develop long-term follow-up pathways and multidisciplinary care models
Surveillance	Case counting alone became insufficient as immunity and variants evolved	Integrate genomic, wastewater, clinical, immunological, and healthcare utilization data

**Legend.** Summary of the principal lessons learned from the COVID-19 pandemic and their implications for future infectious disease preparedness. The table emphasizes that pandemic response should integrate healthcare system resilience, digital infrastructure, scientific collaboration, vaccination strategies, social protection, vulnerable population monitoring, long-term outcome assessment, and multidimensional surveillance.

## Data Availability

No new data were created or analyzed in this study. Data sharing is not applicable to this article.
